# Airborne Dissemination of Bacteria (Enterococci, Staphylococci and *Enterobacteriaceae*) in a Modern Broiler Farm and Its Environment

**DOI:** 10.3390/ani11061783

**Published:** 2021-06-15

**Authors:** Susana Sanz, Carmen Olarte, Raquel Hidalgo-Sanz, Laura Ruiz-Ripa, Rosa Fernández-Fernández, Sara García-Vela, Sandra Martínez-Álvarez, Carmen Torres

**Affiliations:** 1 Tecnología de los Alimentos, Universidad de La Rioja, 26006 Logroño, Spain; carmen.olarte@unirioja.es; 2 Agricultura y Alimentación, Universidad de La Rioja, CCT, C/Madre de Dios, 53, 26006 Logroño, Spain; 3 Bioquímica y Biología Molecular, Universidad de La Rioja, 26006 Logroño, Spain; raquel16196@gmail.com (R.H.-S.); laura_ruiz_10@hotmail.com (L.R.-R.); rosa.fernandez.1995@gmail.com (R.F.-F.); velasarag@gmail.com (S.G.-V.); sandra.martinezal@alum.unirioja.es (S.M.-Á.); carmen.torres@unirioja.es (C.T.)

**Keywords:** air, dissemination, bacteria, broiler farm, *Enterococcus hirae*

## Abstract

**Simple Summary:**

In this study, the density and diversity of relevant groups of bacteria at a broiler farm have been studied, in the inside and outside air and in litter samples. A high number of bacteria was detected in the litter and in the inside air, but a low emission of bacteria was found in the outside air. Moreover, the bacteria detected in the outside air decreased with the distance to the farm. A total of 544 isolates were identified from all the samples (146 from the litter, 142 from inside air and 256 from outside air). From these, 162 staphylococci, 176 *Enterobacteriaceae*, and 190 enterococci were detected. *E. hirae* was the predominant species and the detection of identical DNA profiles in *E. hirae* isolates from inside and outside samples suggests the role of the air in bacterial dissemination from the inside of the broiler farm to the immediate environment. It is necessary to consider the relevance of air as a vehicle of disseminating bacteria at the farm level, which can involve potentially pathogenic bacteria and bacteria carrying antimicrobial resistance genes.

**Abstract:**

The role of the air as a vehicle of bacteria dissemination in the farming environment has been previously reported, but still scarcely studied. This study investigated the bacteria density/diversity of the inside and outside air and of litter samples at a broiler farm. Samples were collected considering two seasons, three outside air distances (50/100/150 m) and the four cardinal directions. Selective media was used for staphylococci, enterococci, and *Enterobacteriaceae* recovery. A high number of bacteria was detected in the litter (2.9 × 10^5^–5.8 × 10^7^ cfu/g) and in the inside air (>10^5^ cfu/m^3^), but a low emission of bacteria was evidenced in the outside air (<6 cfu/m^3^). Moreover, the bacteria detected in the farm’s outside air decreased the further from the farm the sample was taken. A total of 544 isolates were identified by MALDI-TOF (146 from the litter, 142 from inside air and 256 from outside air). From these, 162 staphylococci (14 species; *S. saprophyticus* 40.7%), 176 *Enterobacteriaceae* (4 species; *E. coli* 66%) and 190 enterococci (4 species; *E. hirae* 83%) were detected. *E. hirae* was the predominant species, and identical PFGE clones were detected in inside and outside samples. The detection of identical DNA profiles in *E. hirae* isolates from inside and outside samples suggests the role of the air in bacterial dissemination from the inside of the broiler farm to the immediate environment.

## 1. Introduction

Air plays a key role in the dissemination of some microorganisms, especially molds and viruses, but data about its involvement in the spread of bacteria are still scarce [1]. Airborne particles consist of a mixture of biological material from a range of sources. These particles are generally between 0.3 and 100 µm in diameter, in which microorganisms can appear either as liquid droplets or as dry particles. Smaller particles (ranging in size from 1.0 to 5.0 µm) generally remain in the air and can spread to surrounding fields, while larger particles are deposited on surfaces [2].

Numerous outbreaks associated with the consumption of raw fruits and vegetables have been reported in industrialized countries [3,4,5,6,7]. Vegetable crops can be contaminated with pathogenic/toxigenic bacteria from animal sources through different routes, such as irrigation or manure [7], leading to food poisoning outbreaks when they are ingested either raw or with minimal processing [8,9,10,11,12]. However, these routes do not explain all cases and there is some evidence to support other routes of propagation, in which the air appears to be an additional vehicle of dissemination. This hypothesis is supported by previous works that point towards airborne dissemination of bacteria from farms to neighboring areas, as in the case of cattle farms [13,14], chicken farms [15,16,17,18], and pork farms [19,20].

Intensive poultry production means large densities of animals in small areas, which appears to be a significant source of air pollution [21,22]. This pollution consists of a variety of airborne particles of biological origin in which bacteria are present. These bacteria come from soil, dust feed, litter and from the birds themselves, and may include potential pathogenic bacteria such as enterococci, staphylococci and *Enterobacteriaceae*, among others. The abundance of airborne bacteria varies with the season and location [23].

The majority of studies are focused on the concentration of microorganisms in the air inside poultry houses, but much less is known about the spread of bacteria from fresh litter or inside air to the outside air in surroundings areas [23].

The aim of this study was to assess the bacterial air contamination (especially of potentially foodborne pathogenic enterococci, staphylococci and *Enterobacteriaceae*) in intensive broiler breeding, both inside and outside of the farm, and during two seasons. This work aims to study whether microorganisms from the farm can reach surrounding fields and enter the food chain.

## 2. Materials and Methods

### 2.1. Characteristics of the Broiler Farm

The study was conducted in a modern broiler farm, built in 2015, and located in La Rioja (Spain). The farm covers a total area of 12,000 m^2^ (bounded by a fence), is surrounded by agricultural fields (olive and almond trees to the south, cereal to the west, vineyards to the north and vineyards/non-cultivated area to the east), and is situated 2 km away from the nearest urban area. It consists of two identical and parallel production buildings of 1800 m^2^ area and 3 m height (5400 m^3^) with a north–south orientation. Both buildings are equipped with automatic systems for feeding and watering. The microclimate in the buildings (heating, ventilation, cooling and relative humidity) is managed by an automated computer system: from 33 °C and 65% of relative humidity (RH) at the beginning of the production cycle (2-day flocks) to 19 °C and 85% of RH at the end of the cycle (47-day broilers). Lighting is provided by luminescent lamps for 18 h per day. The buildings are ventilated through a mechanical longitudinal ventilation system with two fans fitted in the south side of the building, five chimneys on the roofs and 80 inlets located in the longitudinal walls (air enters by lateral inlets and is extracted by the fans in the south side). This mechanism expels polluted air from the buildings and draws air into the buildings. Each building has capacity for 31,000 broiler chickens (density 17 broilers/m^2^). The broilers are reared on deep litter (chopped straw 0.10 m thick) and have free access to food and water. The production cycle is 45 days. After the end of each production cycle, manure and litter are cleaned by mobile machinery in both buildings simultaneously. Before loading new flocks of broilers, the buildings are disinfected with a solution of chlorine, NaOH and broad-spectrum insecticide. The disinfectant solution is applied by automatic nebulization and the buildings remain empty for one to two weeks before introducing a new batch.

### 2.2. Sample Collection

Broiler farm sampling was conducted during two different seasons (summer (July 2019) and winter (February 2020)), between 8:00 a.m. and 1:00 p.m. In both cases, it was conducted at the same time in the broiler cycle (15–16 days after chick entry) and temperature and relative humidity inside the building were 27 ± 1 °C and 70%, respectively.

The air samples were taken inside the buildings (inside air) and in the farm surroundings (outside air), at distances of 50, 100 and 150 m in four directions (north, south, east and west) (Figure S1).

Two sampling methods were used at each sampling point: the stationary and mechanical method. In the stationary method, three sticks were firmly set in the middle of each building and in the established sampling points outside. Two culture plates were placed at one-meter height in each stick (six culture plates in each sampling point). The plates were exposed to the air for 4 h. In the mechanical method, a volume of air (100 L of inside air and 1000 L in each outside sampling point) was collected with an Air Ideal air sampler (Biomerieux, Craponne, France). This device allowed the passage of a specific volume of air through a grid with direct impact onto agar plates to facilitate the detection and the count of viable microorganisms. This device takes the air with a constant flow (100 L per minute); thus, it lasted 1 min for the inside sampling and ten minutes for outside sampling.

In addition, samples of litter bed were collected aseptically at the same time and place as the air samples. The free and random movement of the broilers in the enclosure guarantees the homogeneity of the litter bed. The microbiological analysis was performed on 10 g of each litter sample homogenized with 90 mL of sterile peptone water. The homogenized litter samples were subjected to a serial dilution with a dilution factor of 1/10 using a sterile saline (0.9% NaCl) diluent. A volume of 0.1 mL from each decimal dilution was spread onto the surface of agar plates.

Mannitol salt agar (MSA) (Scharlau, Barcelona, Spain), Slanetz-Bartley Agar (SB) (Scharlau, Barcelona, Spain), and Chromocult coliform agar (CCA) (Merck, Darmstadt, Germany) were used for the isolation and enumeration of staphylococci, enterococci and *Enterobacteriacie*, respectively, both from the air and litter samples. Thus, 12 plates were employed for each air sampling point (two of each culture media and two sampling methods). To summarize and taking into account the two sampling seasons: 48 plates for inside air and 288 plates for outside air were used. For litter samples, 6 plates per dilution (two for each culture media) were employed (three dilutions seeded and two sampling seasons made a total of 36 plates).

### 2.3. Bacterial Identification

Up to 10 colonies per plate were randomly taken and grown in brain heart infusion agar (BHIA) (Difco) after 24 h of incubation at 37 °C on selective media. Bacterial morphology was determined by Gram staining and isolates with filamentous morphology and large bacilli (potential *Bacillus*) were excluded from the study. Colonies grown on BHIA were processed for species identification by MALDI-TOF system (matrix-assisted laser desorption/ionization-time of flight) (Bruker Daltonik GmbH, Bremen, Germany), using either direct colony testing or the standard protein extraction protocol, according to manufacturer instructions.

### 2.4. Characterization of Enterococcus hirae Isolates

*E. hirae* was selected for further characterization in order to track the potential dissemination of specific isolates from the inside environment of the farm to the outside air. The antimicrobial resistance phenotype was determined by agar disk diffusion [24] for the following antimicrobial agents (µg/disk): penicillin (10), erythromycin (15), gentamicin (120), tetracycline (30), chloramphenicol (30), linezolid (30), and vancomycin (30). As the genus *Enterococcus* shows an intrinsic low-level resistance for aminoglycosides, in this study, we used disks with a high charge of gentamicin (120 µg/disk) to detect acquired high-level resistance for this antimicrobial [25]. The breakpoints recommended by the Clinical and Laboratory Standards Institute [26] were followed for all antimicrobials. The clonal relatedness of selected *E. hirae* isolates (isolates with similar resistance phenotypes obtained in all three sampling points) was determined by pulsed-field gel electrophoresis (PFGE) of the genomic DNA, after digestion with the endonuclease *Sma*I [27] and PFGE patterns were compared as previously recommended using the GelJ Program [28] and following a previously indicated strategy [29].

## 3. Results

### 3.1. Bacterial Counts

Bacterial counts were calculated with the samples of litter (as cfu/g), and with the samples of inside and outside air obtained with the mechanical method (Air Ideal device) (as cfu/m^3^). Table 1 shows the counts obtained for all the types of samples analyzed, with the observation that in the case of outside air these correspond to the range of all three distances studied (50, 100 and 150 from the farm) and the four cardinal directions (north, south, east and west).

The air inside the farm showed a high bacterial count. In fact, sampling performed with the Air Ideal device (both in summer and winter sampling) programmed to take the smallest possible volume of air (100 L) clogged the plates of all the culture media used (>10^4^ cfu per plate), which indicates counts of over 10^5^ cfu/m^3^.

**Figure 1:**
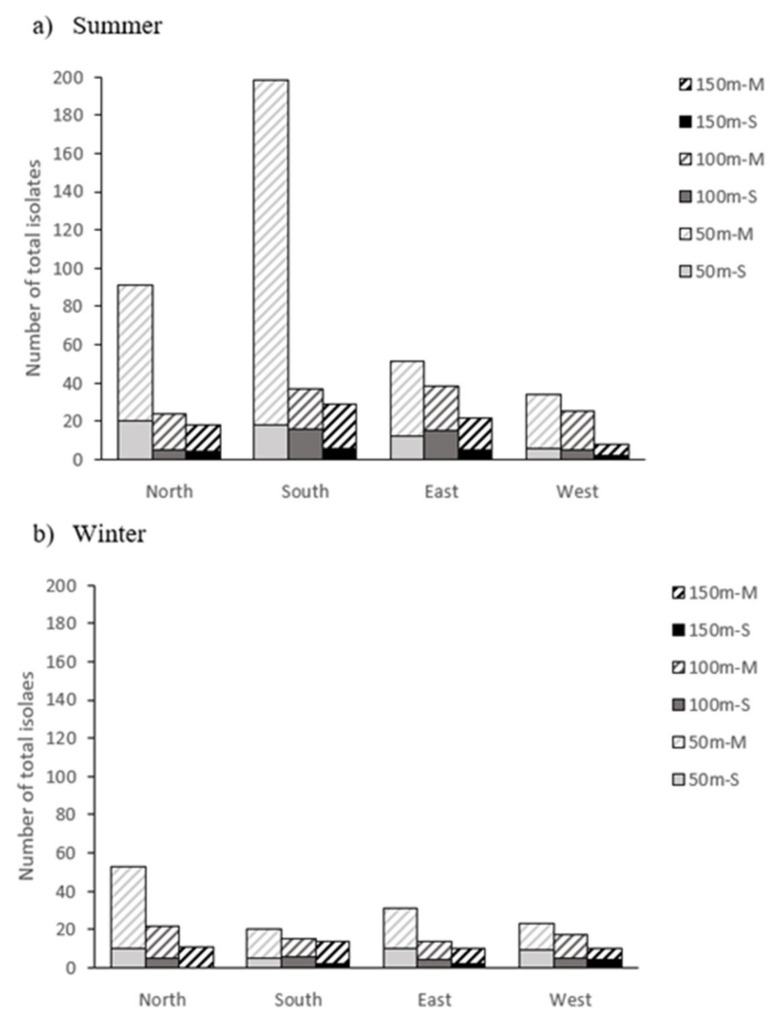
Distribution of total bacterial isolates obtained in the outside air of the broiler farm using both sampling methods (M, mechanical; S, stationary) in relation to the distance (50, 100 and 150 m) from the farm and the cardinal direction in summer (**a**) and winter (**b**).

However, despite the high bacterial load present inside the farm facilities (litter and air), the counts obtained in the outside air were very low: less than 6 cfu/m^3^ in the summer sampling and 4 cfu/m^3^ in the winter sampling. Figure 1 shows the distribution of total isolates obtained in the outside air (*n* = 820) by the two sampling methods used. Of them, 176 isolates were obtained by the stationary sampling method (21%) and 644 by the mechanical sampling method (79%). On the other hand, given that the plates used with the mechanical method were clogged, all the isolates from the inside air were obtained from the stationary method plates.

### 3.2. Diversity of Bacteria Obtained

A total of 1255 isolates were initially obtained (225 from litter, 210 from inside air, and 820 from outside air). Of these, 460 (37%) were eliminated due to morphological reasons (yeasts, micelle structures or presumptive Bacillus colonies). Most of these (79%), were isolated from the MSA medium. The remaining 795 isolates were analyzed using MALDI-TOF and 544 of these were identified at the species level: 146 from litter (65% of the 225 isolates initially obtained), 142 from inside air (67%) and 256 from outside air (30%) (Table 2). The presence of non-identified microorganisms was especially evident in the samples taken from the air outside the farm in the summer sampling. In this sampling, only 137 (53 from MSA, 52 from Chromocult, 32 from SB) of the 575 isolates obtained were identified by MALDI-TOF (23.8%). In the winter sampling, 119 (91 from MSA, 24 from Chromocult, 4 from SB) of the 245 isolates obtained were identified (48.6%).

Table 2 shows the isolates identified from the litter and from the inside and outside air samples. Microorganisms identified in the air outside the farm (*n* = 256) in relation to the sampling point and season are shown in Table 3.

In litter and inside-air samples, *E. hirae* and *E. coli* were the predominant species, accounting for 81% and 70% of the total isolates identified, respectively (43% and 39%, in litter and 38% and 32% in inside air). Regarding the microorganisms identified in the air outside the farm, the predominant species were *S. saprophyticus*, *Pantoea agglomerans* and *E. hirae*, accounting for 60% of total of isolates (20% each). However, *E. coli* was not detected in the outside air.

### 3.3. Characterization of E. hirae Isolates Recovered from Different Sampling Points Showing Similar Resistance Phenotype

*E. hirae* was the predominant specie recovered in this study (29% of total identified isolates), representing 43%, 32% and 19% of those isolates recovered of litter, inside air and outside air, respectively.

The antimicrobial resistance phenotype allowed the classification of the 158 *E. hirae* isolates into six different phenotypic groups: (A) susceptible to all tested antimicrobials (*n* = 94, 59.5%); (B) resistant only to penicillin (*n* = 23, 14.6%); (C) resistant only to tetracycline (*n* = 12, 7.6%); (D) resistant only to erythromycin (*n* = 15, 9.5%); (E) resistant to tetracycline and intermediate resistance to chloramphenicol and susceptible to the remaining antibiotics (*n* = 7, 4.4%); (F) resistant to tetracycline and erythromycin (*n* = 7, 4.4%).

The PFGE profiles showed that two *E. hirae* isolates (phenotypic group E) obtained from litter samples had indistinguishable PFGE patterns with isolates of inside and outside air (obtained at 50 and 150 m away from the farm) (Figure S2a). Identical PFGE profiles were also observed for *E. hirae* isolates of phenotypic group F that were detected in inside air as well as in outside air (at 100 m away from the farm) (Figure S2b).

## 4. Discussion

The strict control of temperature and relative humidity inside the farm, as well as the identical number of animals in the farm, could explain the uniformity of the counts obtained for litter and inside air at both seasons of the year investigated in this study (Table 1). These data correlate with those previously obtained in poultry farms with similar characteristics [19,30,31,32].

The high bacterial load detected in the farm’s inside air could be explained by the fact that it is a closed room in which the environmental conditions (temperature, humidity, daylight absence) remain constant and suitable for the proliferation of microorganisms. The concentration of airborne microorganisms in poultry buildings in other studies varies significantly (between 10^3^ and 10^7^ cfu/m^3^), which could be explained by differences in sampling methods, poultry species (broilers, hens and turkeys), building capacity, density of birds rearing, age of birds and microclimate conditions, among others [16,32].

The farm’s design and management system could explain the very low counts obtained in the outside air, both in summer and winter sampling (Table 1). These low bacterial counts should be attributed to the diluting effect of the air and the stressful conditions of the environment, such as light exposure, temperature changes and dehydration [33]. Previous studies in which the outside air of broiler farms was analyzed (with similar characteristics and sampling methods to our study), also found low densities of bacteria in the air. Kostadinova et al. [32] detected bacterial counts at 2 m away from the farm, only 1 log unit per m^3^ higher than the counts at the control sampling point, located 500 m away from the farm. However, Chinivasagam et al. [16] found values of 10^3^–10^6^ cfu/m^3^, but at 10 m from the ventilation fans. Moreover, the sampling method used affects recovery rates. Schulz et al. [34] and Friese et al. [17,35], using a liquid medium (glass impingers) for the recovery of staphylococci in the air, achieved higher counts (10^6^ cfu/m^3^ and 10^5^ cfu/m^3^, respectively).

In our study, the mechanical sampling method was more effective for the recovery of isolates from the air at low bacterial load conditions (outside air). However, if the bacterial density was very high (inside air), the stationary sampling method was the only one that allowed one to obtain isolates. The use of both simple methods may be adequate to study the microorganisms present in the environment.

It can be clearly observed that the number of isolates was higher in the summer (575) than in the winter (245), and also that the number of isolates decreased with distance from the farm in all cardinal directions. In this sense, the counts were especially high in summer at 50 m south, coinciding with the position of the fans (south side of the buildings), which are often activated at this time. However, this effect was not observed in the counts conducted in winter, which is attributable to the lower activity of the fans in that season. The farm is located on a flat terrain, with no slopes or nearby forest masses. The wind direction changed during sampling periods, and it was not very strong on the days when the sampling was carried out (maximum 10 km/h, both in summer and winter sampling).

The influence of different factors in the distribution of microorganisms in the outside air has already been highlighted by other authors, indicating the importance of the wind in bacterial dissemination, but also highlighting aspects such as the type of animal, the orography and the environmental conditions in the area where the farm is located, as well as the distribution of spaces and the daily activities [16,35,36,37].

Of all the isolates obtained in the samplings carried out, the percentage of isolates identified was very similar for litter and inside air (around 65%). However, only 30% of isolates from the outside air were identified. This percentage is especially high in the summer samplings (more than 75%); this could be explained by a high presence of environmental microorganisms not considered in our study. In fact, identification using MALDI-TOF could not be achieved for 32% of isolates. In most of these unsuccessful cases, the protein spectra obtained did not correspond to any of those listed in the reference library (Biotyper, Bruker), which contains almost all of the bacteria of the relevant groups in this study [38]. It is therefore possible that the unidentified isolates correspond to environmental bacteria that might not have any clinical relevance.

Regarding the microorganisms identified the composition of the microbiota in litter and inside air (Table 2), the results were very similar and in agreement with those reported by other authors [30,39,40], with enterococci and *Enterobacteriaceae* being the major groups. However, in the air outside the farm, in both summer and winter sampling (Table 2 and Table 3), most isolates corresponded to staphylococci and enterococci, although their distribution differed according to the season. Among enterococci and staphylococci, *E. hirae* (76.6%) and *S. saprophyticus* (39.4%) were the predominant species, respectively.

Several authors have already pointed out that both groups of bacteria, but especially staphylococci, have great environmental resistance and are, therefore, those most found in the air. The airborne dissemination of staphylococci has been widely referenced and has even been suggested as an indicator for airborne bacterial emission from animal houses [15,34]. Several studies have shown that enterococci can disseminate from the organic exudates present in the farm to the inside and outside air, leading to the conclusion that air could be an important vehicle for the dissemination of enterococci among different ecosystems [41,42,43].

It is important to note the absence of airborne *E. coli* (both in summer and winter sampling), which contrasts with its high presence in litter and inside air. It has been stated that the survival of *E. coli* is significantly reduced outside, particularly when exposed to direct daylight and high temperatures [18]. Environmental factors such as temperature, relative air humidity, ultraviolet radiation and sampling stress are suggested as factors that cause the low frequencies of the detection of this microorganism in air samples collected outside [44]. However, Laube et al. [18] managed to recover *E. coli* outside a poultry farm using liquid culture media. In a previous study carried out in pork farms, *E. coli* was not found in air samples [20]. The absence of *E. coli* in outside air samples from both pork and broiler farms does not match the results obtained in outside air from cattle farms, where *E. coli* was isolated from many of the samples analyzed [14]. This difference may be due to the type of livestock, since ruminants continuously expel this microorganism (intestinal gases, solid and liquid fecal matter), unlike other animals that produce fewer emissions.

Regarding the characterization of *E. hirae* isolates, and although this work did not aim to study the antibiotic resistance of the isolates obtained, it is important to highlight that 40.5% of *E. hirae* isolates were resistant to at least one of the antimicrobial agents evaluated. The presence of antimicrobial-resistant enterococci and staphylococci is a growing problem, and their airborne dissemination has been analyzed in several works. Recent studies revealed the airborne exchange of antimicrobial-resistant bacteria from livestock farms to the environmental microbial community [18,35,37,45].

The detection of identical genomic DNA profiles in *E. hirae* isolates recovered from litter, inside and outside samples leads to the conclusion that the air was involved in bacterial dissemination from the inside of the broiler farm to the immediate environment.

## 5. Conclusions

In modern broiler farms, the emissions of bacteria to distances greater than 50 m is very low, despite the large number of bacteria in the inside air.

Although the bacterial exchange does not seem quantitatively significant, the finding of bacteria with the same genetic profiles in the interior and in the air 150 m away shows that this exchange exists.

Thus, it is necessary to consider the relevance of air as a vehicle of bacterial dissemination at the farm level, which can involve potentially pathogenic bacteria. Similarly, the possible airborne dissemination of bacteria carrying antimicrobial resistance genes should be monitored.

## Figures and Tables

**Table 1:** Bacterial counts obtained with the mechanical method, in the different types of samples (inside and outside air and litter), in different types of media with indication of sampling conditions.

Sample	Season	Sampling Conditions		Bacterial Counts in the Culture Media		
		T (°C)	RH (%)	MSA	SB	CCA
Litter ^a^	Winter	26–28	70–72	4.9 × 10^7^ cfu/g	2.6 × 10^6^ cfu/g	2.9 × 10^5^ cfu/g
	Summer	26–28	70–72	5.8 × 10^7^ cfu/g	4.7 × 10^6^ cfu/g	3.1 × 10^5^ cfu/g
Inside air ^b^	Winter	26–28	70–72	>10^5^ cfu/m^3^	>10^5^ cfu/m^3^	>10^5^ cfu/m^3^
	Summer	26–28	70–72	>10^5^ cfu/m^3^	>10^5^ cfu/m^3^	>10^5^ cfu/m^3^
Outside air ^c^	Winter	2.4–14.7	53–77	n.d.−4 cfu/m^3^	n.d.−3 cfu/m^3^	n.d.−2 cfu/m^3^
	Summer	17.1–29.0	42–68	n.d.−6 cfu/m^3^	n.d.−4 cfu/m^3^	n.d.−5 cfu/m^3^

^a^ The bacterial counts in the litter are the average values. ^b^ The bacterial counts in the inside air are estimated because of the high bacterial load in this samples. ^c^ The bacterial counts in the outside air correspond to the range of results obtained at all three distances (50, 100, 150 m from the farm) and the four cardinal directions (north, south, east and west). n.d.: no bacterial growth in the plate, T: Temperature, RH: Relative humidity; MSA: Mannitol salt agar, SB: Slanetz-Bartley, CCA: Chromocult coliform agar.

**Table 2:** Distribution of isolates identified in litter and in the inside and outside air of the sampled broiler farm (the percentage of each bacterial group over the total of each type of sample is indicated in parentheses).

Microorganisms:	Air		
	Litter	Inside	Outside
***Staphylococci:***	**15 (10%)**	**20 (14%)**	**127 (50%)**
*Staphylococcus aureus*	4	-	-
*S. arlettae*	-	1	19
*S. capitis*	-	-	1
*S. cohnii*	-	1	5
*S. epidermidis*	1	-	6
*S. equorum*	-	-	6
*S. haemolyticus*	-	-	1
*S. hominis*	-	-	6
*S. lentus*	1	3	2
*S. nepalensis*	-	1	4
*S. pasteuri*	1	-	-
*S. saprophyticus*	6	10	50
*S. sciuri*	-	-	1
*S. xylosus*	2	4	26
***Enterobacteriaceae:***	**57 (39%)**	**68 (48%)**	**51 (20%)**
*Escherichia coli*	57	54	-
*Escherichia hermani*	-	-	1
*Klebsiella pneumoniae*	-	11	-
*Pantoea agglomerans*	-	3	50
***Enterococci:***	**74 (51%)**	**52 (38%)**	**64 (25%)**
*Enterococcus faecalis*	8	2	3
*E. faecium*	3	4	10
*E. hirae*	63	46	49
*E. casseliflavus*	-	-	2
**Others:**	**-**	**2**	**14 (5%)**
*Achromobacter spanius*	-	-	1
*Acinetobacter radioresistens*	-	1	3
*Acinetobacter baumanii*	-	1	-
*Aerococcus viridans*	-	-	6
*Corynebacterium proping.*	-	-	1
*Micrococcus luteus*	-	-	1
*Pseudomonas corrugata*	-	-	1
*Pseudomonas koreensis*	-	-	1
**TOTAL**	**146**	**142**	**256**

**Table 3:** Distribution of isolates identified in the outside air of the sampled broiler farm in relation to the point of sampling (distance from the farm and cardinal direction).

Microorganisms (Number in Summer/Winter)	North			South			East			West		
	50 m	100 m	150 m	50 m	100 m	150 m	50 m	100 m	150 m	50 m	100 m	150 m
***Staphylococci:* 24/103**	**5/28**	**1/8**	**-/5**	**6/5**	**4/2**	**2/3**	**-/14**	**-/3**	**1/7**	**4/20**	**1/7**	**-/1**
*S. arlettae*	-/2	-/1	-/1	3/-	-	-	-/2	-	-/1	-/8	-/1	-
*S. capitis*	-	-	-	-	1/-	-	-	-	-	-	-	-
*S. cohnii*	-/1	-	-	-	-	-	-/2	-	-	-/1	-	-/1
*S. equorum*	-/1	-	-	-/4	-/1	-	-	-	-	-	-	-
*S. epidermidis*	4/1	-	-	-	1/-	-	-	-	-	-	-	-
*S. haemolyticus*	-	-	-	-	-	-/1	-	-	-	-	-	-
*S. hominis*	-	-/1	-	-	1/1	-/2	-	-	-	1/-	-	-
*S. lentus*	-	-	-	-	-	-	-/1	-	-/1	-	-	-
*S. nepalensis*	-	-	-	-	-	-	-	-	-	-/4	-	-
*S. saprophyticus*	-/19	1/6	-	2/-	1/-	-	-/9	-/3	1/-	-/7	-/1	-
*S. sciuri*	-	-	-	-	-	1/-	-	-	-	-	-	-
*S. xylosus*	1/4	-	-/4	1/1	-	1/-	-	-	-/5	3/-	1/5	-
***Enterobacteriaceae:* 46/5**	**4/2**	**6/-**	**4/-**	**1/-**	**4/1**	**-**	**-**	**11/1**	**4/-**	**9/1**	**3/-**	**-**
*Escherichia hermani*	-	-	-	-	1/-	-	-	-	-	-	-	-
*Pantoea agglomerans*	4/2	6/-	4/-	1/-	3/1	-	-	11/1	4/-	9/1	3/-	-
***Enterococci:* 58/6**	**6/-**	**2/-**	**2/-**	**13/-**	**7/-**	**-**	**10/-**	**1/5**	**4/-**	**13/1**	**-**	**-**
*E. faecalis*	-	-	-	-	3/-	-	-	-	-	-	-	-
*E. faecium*	2/-	1/-	-	1/-	1/-	-	1/-	-/2	-	1/1	-	-
*E. hirae*	4/-	-	2/-	12/-	3/-	-	9/-	1/3	3/-	12/-	-	-
*E. casseliflavus*	-	1/-	-	-	-	-	-	-	1/-	-	-	-
**Others: 9/5**	**-/1**	**2/-**	**1/-**	**-**	**1/-**	**2/-**	**1/1**	**-/2**	**2/1**	**-**	**-**	**-**
*Achromobacter spanius*	-/1	-	-	-	-	-	-	-	-	-	-	-
*Acinetobacter radioresistens*	-	1/-	-	-	-	-	-	-	2/-	-	-	-
*Aerococcus viridans*	-	-	-	-	1/-	2/-	-	-/2	-/1	-	-	-
*Corynebacterium propinq.*	-	-	-	-	-	-	-/1	-	-	-	-	-
*Micrococcus luteus*	-	1/-	-	-	-	-	-	-	-	-	-	-
*Pseudomonas corrugata*	-	-	1/-	-	-	-	-	-	-	-	-	-
*Pseudomonas koreensis*	-	-	-	-	-	-	1/-	-	-	-	-	-

## Data Availability

Not applicable.

## Supplementary Materials

The following are available online at https://www.mdpi.com/article/10.3390/ani11061783/s1. Figure S1. location of sampling points (red dots). The dashed line indicates the fence that delimits the farm. Figure S2. (**a**): PFGE patterns of the seven *E. hirae* isolates of phenotypic group E (resistance to tetracycline and intermediate resistance to chloramphenicol) obtained from different origins. Lanes 1, 2 and 3: isolates from litter; Lane 4 and 5: isolates from inside air; Lane 6 and 7: isolates from outside air (50 m west and 150 m east, respectively). Lanes 2, 3, 4, 6 and 7 show indistinguishable banding pattern. (**b**): PFGE patterns of the seven *E. hirae* isolates of phenotypic group F (resistance to tetracycline and erythromycin) isolated from different origins. Lane 1 and 2: isolates from litter; Lane 3 and 4: isolates from inside air; Lane 5 and 6 isolates from outside air in summer sampling (100 m south and 100 m east, respectively); Lane 7: isolate from outside air in winter sampling (100 m east). Lanes 3, 4, 6 and 7 show indistinguishable banding pattern.
