# Supplementary material for: Airborne Dissemination of Bacteria (Enterococci, Staphylococci and Enterobacteriaceae) in a Modern Broiler Farm and Its Environment
Source: Animals (Basel). 2021 Jun 15;11(6):1783. doi: 10.3390/ani11061783 (PMC8232102; doi:10.3390/ani11061783)

animals

Article

Airborne dissemination of bacteria (enterococci, staphylococci and *Enterobacteriaceae*) in a modern broiler farm and its environment

Susana Sanz[1*], Carmen Olarte[1], Raquel Hidalgo-Sanz[2], Laura Ruiz-Ripa[2], Rosa Fernández-Fernández[2], Sara García-Vela[2], Sandra Martínez-Álvarez[2] and Carmen Torres[2]

1 Tecnología de los Alimentos, Universidad de La Rioja, Logroño. Spain
2 Bioquímica y Biología Molecular, Universidad de La Rioja, Spain
* Susana Sanz. Dpto. Agricultura y Alimentación. Universidad de La Rioja. CCT, C/Madre de Dios, 53. 26006 Logroño (La Rioja) Spain. e-mail: susana.sanz@unirioja.es Phone number: +34 941299729 Fax number: +34 941299721

Supplementary Materials: The following are available online at www.mdpi.com/xxx/s1,

Figure S1: Location of sampling points (red dots). The dashed line indicates the fence that delimits the farm.

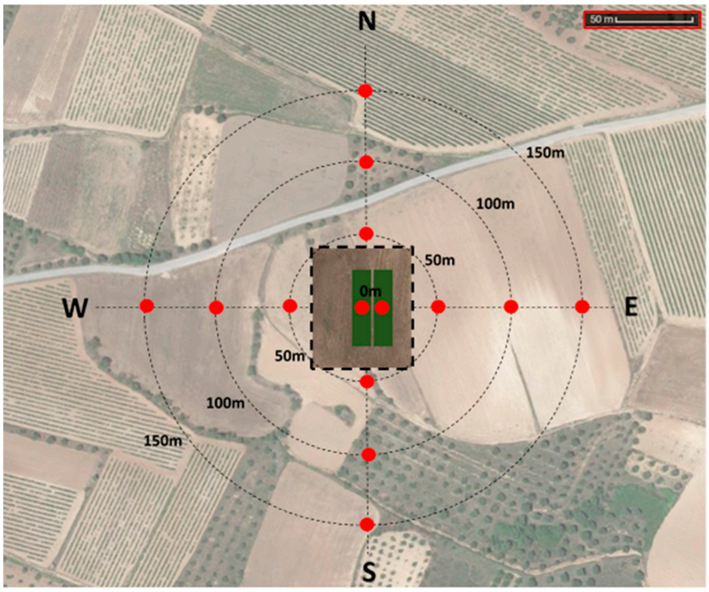

Figure S2a: PFGE patterns of the seven *E. hirae* isolates of phenotypic group E (resistance to tetracycline and intermediate resistance to chloramphenicol) obtained from different origins. Lanes 1, 2 and 3: isolates from litter; Lane 4 and 5: isolates from inside air; Lane 6 and 7: isolates from outside air (50 m west and 150 m east, respectively). Lanes 2, 3, 4, 6 and 7 show indistinguishable banding pattern.

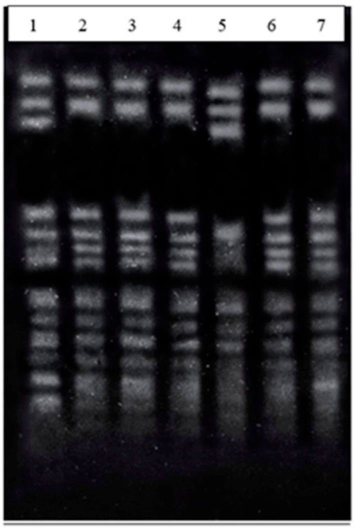

Figure S2b: PFGE patterns of the seven *E. hirae* isolates of phenotypic group F (resistance to tetracycline and erythromycin) isolated from different origins. Lane 1 and 2: isolates from litter; Lane 3 and 4: isolates from inside air; Lane 5 and 6 isolates from outside air in summer sampling (100 m south and 100 m east, respectively); Lane 7: isolate from outside air in winter sampling (100 m east). Lanes 3, 4, 6 and 7 show indistinguishable banding pattern.

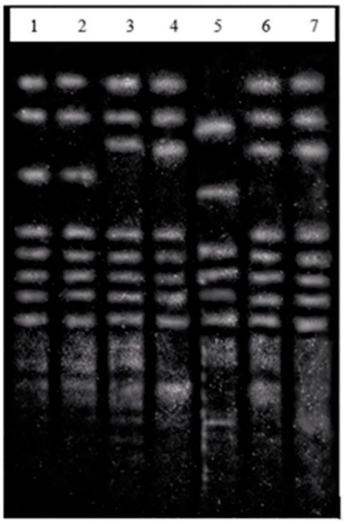

Supplement: Supplementary file 1 [file animals-11-01783-s001.zip › animals-1262251-supplementary.pdf]
